# Using the Idexx SediVue Dx to predict the need for urine bacteriologic culture in cats

**DOI:** 10.1177/10406387211038918

**Published:** 2021-08-17

**Authors:** Elisabeth Neubert, Karin Weber

**Affiliations:** Clinic of Small Animal Medicine, Centre for Clinical Veterinary Medicine, Ludwig-Maximilians-Universitaet Munich, Munich, Germany

**Keywords:** bacteriuria, cats, clinical laboratory test, pyuria, veterinary

## Abstract

We analyzed urine samples from 191 cats for bacteriuria with an automated urine sediment analyzer (Idexx SediVue Dx), combined with image review by an observer, and compared to bacteriologic culture results. Sixty-nine samples were unambiguously assigned to be free of bacteria by the instrument and the observer, and no bacterial growth was detected. Twenty-seven samples were unambiguously assigned to have bacteriuria; 24 of these 27 samples were culture-positive. For these samples, bacteriuria was predicted with a sensitivity of 100% and a specificity of 96%. A clear assignment was not possible for 95 samples, 81 of which were culture-negative. Specificity dropped to 45% when all samples were considered. Using the automated leukocyte count to predict bacteriuria, sensitivity was 82% and specificity was 75%. Automated sediment analysis is faster and less observer-dependent than sediment analysis under a microscope, but accurate detection of bacteriuria remains difficult in a large proportion of samples. Bacteriuria was significantly associated with leukocyte count; the leukocyte count was >5/high power field in 82% of culture-positive samples.

Complete urinalysis, including sediment analysis, is considered a core laboratory test for dogs and cats in small animal practice.^8^ The accurate detection of bacteriuria is important to decide which samples need to be tested further with quantitative bacteriologic cultures including an antibiogram, and which patient might benefit from an immediate start of antimicrobial therapy while culture results are pending. Superfluous laboratory tests should be avoided from an economic point of view, and needless administration of antimicrobials is not consistent with antimicrobial stewardship and may elicit adverse effects.

Urine sediment analysis via light microscopy is highly dependent on the experience of the technician, and in an unstained preparation, amorphous crystals and cell debris are frequently mistaken as bacteria.^2^ The detection of bacteria is improved considerably in air-dried and Gram- or Wright-stained preparations, but this adds further hands-on time.^7^ The use of instruments for automated urine sediment analysis is increasing in veterinary clinics to eliminate several sample preparation steps for faster results and better comparability between observers.^4^ One of these instruments is the Idexx SediVue Dx, which combines urine centrifugation, automatic sediment analysis, and image capturing of the unstained sediment with a built-in camera. Sediment particles are analyzed and classified by algorithms, but the operator is advised to always review the images. We assessed the reliability of automated analysis of cat urine samples for the detection of bacteria by comparing the automated results of the SediVue, the results of the image review by the operator, and the results of bacteriologic cultures.

In this retrospective study, we searched the patient database of the Clinic of Small Animal Medicine, Ludwig-Maximilians-University (LMU; Munich, Germany) for cats with a complete urinalysis, including a sediment analysis with the Idexx SediVue Dx and bacteriologic examination with an antibiogram, from January 1–December 31, 2018. The database search yielded 303 datasets from 219 cats with bacteriologic examination and urinalysis. All datasets were screened for consistency and completeness to ensure that the bacteriology result belonged to the correct urine sample. We rejected 112 datasets because of missing data (bacteriologic examination of sample other than urine; urine sample type not recorded; sediment not analyzed with the SediVue), leaving 191 samples from 168 individual cats. Two samples were analyzed from 17 cats, and 4 or more samples were analyzed from 6 cats.

The SediVue automatic analysis for bacteriuria classifies rods and cocci with 3 grades (“none to rare,” “suspect present,” or “present”). Digital images of the sediment are archived, and the workflow for the detection of bacteriuria includes the evaluation of these images by a human observer. In our study, the observer was a veterinary student in the clinical semester who was trained with sediment sample images before evaluating the datasets. For each complete dataset, the observer classified the archived images the same as the SediVue: rods or cocci were either “none to rare,” “suspect present,” or “present” in the images. The observer was blinded to the results of the bacteriologic examination but could use the SediVue results according to the workflow defined in the instruction manual for the instrument. The results of the bacteriologic examination were recorded with the type of organism and number of cfu/mL. The threshold for a potentially clinically relevant amount of bacterial growth was set at >10^3^ cfu/mL for all urine samples. All samples had been refrigerated continuously and were cultured within 24 h on campus by the Institute of Infectious Diseases and Zoonoses, Faculty of Veterinary Medicine, LMU.

Sex, breed, urine collection method (cystocentesis, catheter, or voided), and urine leukocyte count as reported by the SediVue (5 categories: 0, 1–5, 6–20, 21–50, >50 WBC/hpf) were also included in the data table. Of the 191 datasets evaluated, 38 samples (20%) had bacterial growth of >10^3^ cfu/mL (Table 1). Only 2 samples had mixed bacterial growth >10^3^ cfu/mL for 2 organisms. Bacterial growth up to 10^3^ cfu/mL was found in 6 samples (3%). In 69 samples, the instrument and the observer classified the sediment to be free of bacteria and rated “none to rare” for both rods and cocci. In none of these samples was bacterial growth detected above the cutoff. Most of these samples also had very low leukocyte counts (Table 1).

**Table 1.** Results for the detection of bacteria by both automated sediment analysis and the observer compared to bacteriologic culture and leukocyte count.

	Culture-positive	Culture-negative	0–5 WBC/hpf	6–20 WBC/hpf	>20 WBC/hpf
Bacteria “present”	24	3	4 (3/1)	3 (3/0)	20 (18/2)
Bacteria “none to rare”	0	69	63 (0/63)	5 (0/5)	1 (0/1)
Bacteria “suspect present”	14	81	55 (4/51)	26 (2/24)	14 (8/6)

Culture-positive/culture-negative in parentheses. Culture-positive = bacterial growth >10^3^ cfu/mL.

In 27 samples, the instrument and the observer both classified bacteria to be present in the sediment; bacteria were cultured from 24 of these samples. In 22 samples, the instrument and the observer both classified rods to be present in the sediment. Bacteria were cultured (>10^3^ cfu/mL) from 20 samples, yielding *Escherichia coli* in 13 samples, *E. coli* and *Streptococcus canis* in 1 sample, *E. coli* and *Enterococcus faecalis* in 1 sample, *Proteus mirabilis* in 3 samples, *Enterobacter cloacae* in 1 sample, and *S. canis* in 1 sample. Most of these samples had high leukocyte counts, with >20 WBC/hpf in 17 samples, including the 2 samples with no bacterial growth. A moderate leukocyte count was found in 3 samples and a low leukocyte count in 2 samples.

In 5 samples, the instrument and the observer both classified cocci to be present in the sediment. Bacteria could be cultured (>10^3^ cfu/mL) in 4 samples, yielding *Staphylococcus felis* in 1 sample, *S. pseudintermedius* in 1 sample, and *E. coli* in 2 samples. A high leukocyte count was found in 3 of the 4 bacteria growth–positive samples. Both the positive and negative predictive values (PPV, NPV) were high if the instrument and the observer came to the same result (Table 2).

**Table 2.** Results of bacterial culture for samples that were classified by both the Idexx SediVue and the observer to be either positive (bacteria “present”) or negative (bacteria “none to rare”) for bacteria.

	Culture-positive	Culture-negative	Total	
Bacteria “present”	24	3	27	PPV 89%
Bacteria “none to rare”	0	69	69	NPV 100%
Total	24	72	96	

Culture-positive = bacterial growth >10^3^ cfu/mL; NPV = negative predictive value; PPV = positive predictive value; sensitivity = 100% (95% CI: 86–100%); specificity = 96% (95% CI: 88–99%).

In the remaining 95 samples (50%), the classification of the instrument and of the observer did not agree, and all combinations of “none to rare,” “present,” and “suspect present” occurred. Bacterial growth above the threshold was detected in 14 of these samples (7 samples with *E. coli*, 3 with *Enterococcus* spp., and 1 each with *Enterobacter* spp., *Moraxella, Yersinia pseudotuberculosis*, and *Staphylococcus epidermidis*). The ratings for the (suspected) presence or absence of rods and cocci diverged frequently and were not reliable as a predictor of bacterial growth. Disagreement was higher for cocci (91 samples) than for rods (41 samples). Twenty-nine samples were classified by the instrument as bacteria “none to rare” and as “present” by the observer, with 6 culture-positive samples. Twenty-three samples were classified by the observer as bacteria “none to rare” and as “present” by the instrument, with 8 culture-positive samples (Table 3).

**Table 3.** Results of bacterial culture for all samples in which “bacteria detected” rods or cocci were classified “present” or “suspect present” by the Idexx SediVue or the observer.

	Culture-positive	Culture-negative	Total	
Bacteria detected	38	84	122	PPV 31%
Bacteria “none to rare”	0	69	69	NPV 100%
Total	38	153	191	

Culture-positive = bacterial growth >10^3^ cfu/ml; NPV = negative predictive value; PPV = positive predictive value; sensitivity = 100% (95% CI: 91–100%); specificity = 45% (95% CI: 37–53%).

Leukocytes in urine are associated with urinary tract infections but may also be seen in inflammatory processes without underlying bacterial infection. Significant bacterial growth was found in only 6% of the samples with a leukocyte count ≤5 WBC/hpf (Table 4). We also analyzed the parameter WBC for the 95 samples that were assigned bacteria “suspect present” to assess whether it could help to find the culture-positive samples, but the PPV was low (Table 5).

**Table 4.** Results of bacterial culture for all samples using the Idexx SediVue leukocyte count.

Leukocyte count	Culture-positive	Culture-negative	Total	
>5 WBC/hpf	31	38	69	PPV 45%
≤5 WBC/hpf	7	115	122	NPV 94%
Total	38	153	191	

Culture-positive = bacterial growth >10^3^ cfu/mL; NPV = negative predictive value; PPV = positive predictive value; sensitivity = 82% (95% CI: 66–92%); specificity 75% (95% CI: 68–82%).

**Table 5.** Results of bacterial culture for samples classified as bacteria “suspect present” using the Idexx SediVue leukocyte count.

Leukocyte count	Culture-positive	Culture-negative	Total	
>5 WBC/hpf	10	30	40	PPV 25%
≤5 WBC/hpf	4	51	55	NPV 93%
Total	14	81	95	

Culture-positive = bacterial growth >10^3^ cfu/mL; NPV = negative predictive value; PPV = positive predictive value; sensitivity = 71% (95% CI: 42–92%); specificity = 63% (95% CI: 52–73%).

When urinalysis is not restricted to patients with signs of urinary tract infection, the percentage of samples without any bacterial growth after cultivation is high. In our study, only 20% of samples had a potentially clinically relevant amount of bacterial growth. Variable ranges were seen in previous studies in dogs (16% of all samples showed bacterial growth), cats (6–29%), and humans (2.3–29%).^3,5-7,9,10^ Predicting culture-negative samples by sediment analysis would lead to fewer additional laboratory tests, and antimicrobial therapy would not be considered for these patients. In our study, when both the automatic algorithm of the SediVue and the image review by the human observer did not detect any bacteria, no relevant number of bacteria could be cultivated. For these samples (36% of all samples), the bacteriologic examination did not add any relevant information.

Detection of bacteriuria in canine and feline urine sample can be difficult, and accuracy depends on the method. Cocci are especially difficult to distinguish from amorphous particles (pseudobacteria) in an unstained wet-mount. These detection difficulties were reflected in our study in the frequent classification of “suspect present” for cocci, both by the automatic algorithm and the human observer. In samples with the opposing results of bacteria “none to rare” or bacteria “present” depending on the method, neither instrument nor human observer appeared to be superior in predicting the outcome.

One sample was classified as “rods present” but yielded *S. canis* growth. Cocci are often attached to each other in short chains, which may resemble rods. Three samples did not have bacterial growth although both the instrument and the observer agreed on the presence of bacteria. This may be because of previous antimicrobial therapy that stopped bacterial proliferation, or to a bacterial strain such as *Corynebacterium urealyticum* that would not grow using routine urine cultures.^1^

Wright staining of dried sediment considerably improves sensitivity and specificity of bacterial detection compared to the microscopic examination of an unstained wet-mount, but adds hands-on time.^7,9,10^ Sensitivity of Wright-stained dried sediment of 82.8% and specificity of 98.6% have been reported compared with culture results in cat urine, which is considerably higher specificity than with the automated detection method of our study.^9^ However, compared to a wet-unstained examination under the microscope, the sensitivity of our automated method appears to be higher (100% vs. 75.9%), and the specificity slightly lower (45% vs. 57.8%).^9^

In a previous study, the leukocyte count was high in only 34% of cat urine samples with a positive urine culture, which is in contrast to our study, in which 82% of the samples with a positive urine culture had leukocyte counts >5 WBC/hpf.^9^ In another study, leukocyte counts >5 WBC/hpf were found in 46% of culture-positive samples from dogs and in 57% of culture-positive samples from cats.^7^ In a large study in humans with 758 culture-positive samples and automatic sediment analysis, 68% of the samples had a leukocyte count ≥5 WBC/hpf, with sensitivity of 68%, specificity of 88%, PPV of 12%, and NPV of 99% for bacteriuria.^5^ The cutoffs for leukocytes and pyuria differ, as does the definition of a high-power field and the preanalytical treatment of samples, which makes comparisons of the parameter leukocyte count between studies difficult. However, the sensitivity of the automated WBC count for bacteriuria appears to be comparable to the analysis of stained dry sediment in cat urine (82% vs. 82.8%), although the specificity is lower (75% vs. 98.7%).^9^ For the 95 samples that were assigned bacteria “suspect present,” the parameter WBC did not help in predicting bacteriuria, with a low PPV of 25%. A display of the leukocyte count in absolute numbers instead of categories on the SediVue might be helpful to find a threshold for both acceptable sensitivity and specificity. A limitation of our study is that we could not include a control method (dried and stained sediments) in our retrospective study.

Automated sediment analysis, together with a review of the images, helps to quickly classify samples that would not need further analysis, or which show obvious bacteriuria and are always sent to a microbiology laboratory. However, for ~50% of the samples, a conclusion of bacteriuria yes or no could not be reached by automated sediment analysis. These samples could either be centrifuged, dried, and stained manually to reach a conclusion on the same day, or they could be sent for urine culture, with results 1–2 d later.
